# A Novel Class of Functionalized Synthetic Fluoroquinolones with Dual Antiproliferative - Antimicrobial Capacities

**DOI:** 10.31557/APJCP.2021.22.4.1075

**Published:** 2021-04

**Authors:** Abdelrahman Al-Nuaimi, Yusuf Al-Hiari, Violet Kasabri, Randa Haddadin, Noor Mamdooh, Sundus Alalawi, Sara Khaleel

**Affiliations:** 1 *School of Pharmacy, University of Jordan, Amman 11942, Queen Rania Street, Jordan. *; 2 *Zarqa Private University, Zarqa, Jordan.*

**Keywords:** Quinolones, Fluoroquinolones, Triazoloquinolones, Sulphorodhamine B, Cisplatin and cancer

## Abstract

As vosaroxin as a fluoroquinolone (FQ) had anticancer effectiveness; this study aimed to screen new lipophilic FQs for their dual antimicrobial-antiproliferative activities. Using sulforhodamine B assay; 36 lipophilic FQs have been screened for antimicrobial propensities against* S. aureus*, *E. coli*, and *C. albicans* vs. the respective references ciprofloxacin and fluconazole. They were also explored against a battery of cancer cell lines. Normal periodontal ligament fibroblasts (PDL) were tested for safety examination in comparison to the cisplatin. Reduced FQ compound 4g (R-2, 4-DMeOACA) highly scored nanomolar potency with MIC value of 0.004 µM against gram-positive bacteria. The highest activity of the 36 lipophilic FQs was noted on Leukaemia K562, cervical HELA and pancreatic PANC-1 cancer cell lines with respective IC_50_ value of 0.005 µM for compound R-4-BuACA (4e), 0.40 µM with CHxCA (7a) and 0.11 µM for R-4-HxACA (4f). Tested FQs exhibited cytotoxicity in A549 lung cancer, MCF-7 and T47D breast cancer cell lines. The reduced 4e and 4f compounds have shown nanomolar inhibition against K562 (as of 4e), PANC-1 and MCF-7 (as of 4f) with IC50 values of 0.005, 0.11 and 0.30 µM, respectively. Succinctly FQs’ dual gram-positive antibacterial-antineoplastic capacities expand on of drug design scaffolds in lead generation.

## Introduction

The fight against cancer is an unbreakable continuous challenge. Cancer statistics are overwhelming and new cases are arising each year. The cost, resistance and serious side effects of existing therapy made such battle more complicated. The need for new cheaper agents with new mechanism or lesser side effects is substantiated urgently especially to overcome resistance issues. FQs as new anticancer agents might be the answer for such problems (Mondal et al., 2004; Degu et al., 2017). Antibacterial quinolones have been used since 1960s to treat the pulmonary, urinary and other infectious disease. Ciprofloxacin, gemifloxacin, levofloxacin and moxifloxacin are among antibacterial FQs (Drlica, 1999). Several reports have mentioned that FQs have anti-proliferative activity in certain types of human cancer cell lines such as moxifloxacin and ciprofloxacin and gemifloxacin (Kan et al., 2013). In 2013, the drug vosaroxin was a groundbreaking addition to anticancer approach based on quinolones. Vosaroxin and all anticancer quinolone do inhibit eukaryotic type II topoisomerases (A2) that is highly expressed in many eukaryotic proliferating cells (Abbas et al., 2015; Hotinski et al., 2015). This anticancer FQ drug does justify and rationalize our aims toward antineoplastic FQs. 

Additional rationalization for this research comes from the fact that FQs class was in general regarded as quite safe overall, without any significant cardio toxicity (Owens and Ambrose, 2005). Moreover, quinolones have superior physicochemical properties with excellent bioavailability and pharmacokinetics thus efflux-based resistance might not emerge as a major issue (Bisacchi and Hale, 2016). Quinolones are easily formulated as both oral and parenteral preparations.

**Figure 1:**
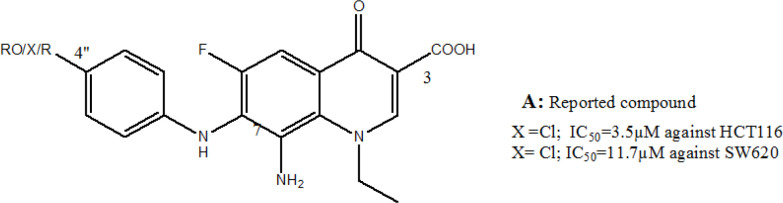
New FQs by Our Group and Their IC_50_ against Colon Cancer Cells (Arabiyat et al. 2016a,b; 2017; Alabsi et al. 2018; Jumah 2018; AlShahrabi et al., 2020)

Our group has started recently a research line focusing on preparing novel lipophilic FQ derivatives with noticeably promising antiproliferative activity against human colon cancer cell lines (Arabiyat et al., 2016a,b; 2017; Alabsi et al., 2018; Jumah, 2018; AlShahrabi et al., 2020). Our previous work with the 6-fluoro-8-nitro-4-oxo-1,4-dihydroquinoline-3-carboxylic acid FQs scaffold have spotted the light on lipophilicity and size as most important requirements for appreciable antiproliferative FQ. Therefore, this work aims at testing same lipophilic FQs prepared previously by our group (Arabiyat et al., 2016a,b; 2017; Alabsi et al., 2018; Juma, 2018; AlShahrabi et al., 2020) against new cell lines including Malignant Melanoma A375; Leukaemia K562; breast T47D and MCF7 cancer and cervical cancer cell HELA lines; Lung cancer cell A549 and Pancreatic1 (Panc-1) and normal periodontal ligament fibroblasts (PDL) for safety examination in comparison to the cisplatin. The lipophilicity was imposed on these structures through substitution with halogenated aniline at C-7 and N-ethyl at N1, (Figure 1).

## Materials and Methods

All reagents and chemicals of analytical grade were obtained from Sigma (Dorset, UK); unless stated otherwise. All chemicals, reagents and solvents (of analytical grade) were used directly without further purification. MCF7 cell line ATCC^®^ HTB-22™, T47D cell line ATCC^®^ HTB-133™, K562 cell line ATCC^®^ CCL-243™, Panc1 cell line ATCC^®^ CRL-1469™. A375.S2 cell line ATCC^®^ CRL-1872™, A549 cell line ATCC^®^ CCL-185™, HELA cell line ATCC® CCL-2™ were cultured in Dulbecco’s modified eagle’s medium (DMEM). (DMEM) containing foetal bovine serum (FBS) (Bio Whittaker, Verviers, Belgium), 4-(2-hydroxyethyl)-1-piperazine ethane sulfonic acid (HEPES) Buffer, L-glutamine, gentamicin, penicillin, and streptomycin sulphate (Sigma, St. Luis, MO, USA), trypsin, trypan blue, Ciprofloxacin HCl from Aarti drugs Ltd/India, was a kind gift from Pharma International Company- Amman; Fluconazole from Neopharma, United Arab Emirates, a kind gift from Pharmaceutical Research Unit, Amman; Trypon soya agar (TSA), trypon soya broth (TSB), sabouraud dextrose agar (SDA) were obtained from Liofilchem (Italy) or Oxoid LTD (Basingstoke, England), they were reconstituted and sterilized by autoclave at 121^°^C for 15 min.

*General procedure to prepare target compounds* scheme 1 *,* 2 *,* 3

The spectral data of all compounds were reported elsewhere (Arabiyat et al. 2016a,b; 2017;Alabsi et al. 2018; Jumah 2018; AlShahrabi et al., 2020). All compounds are tested for different activities and the chemistry is sent for publication with full spectral data. Some spectral and elemental data are provided as supplementary.

*a- Nitro ester derivatives 2a-g, 6s, 10a-c*

Three molar equivalents of aniline (3M excess) were added into a solution containing 1Mole of (1E in scheme 1 and 2 or 1EtE in scheme 3) and 10-15 mL of DMSO as a solvent and drops of pyridine then was refluxed at 65-70^°^C under anhydrous conditions for (4-5) days. The reaction mixture was monitored until no starting material remained then was left to crystallize at room temperature. The product was filtered, left to dry in dark place. Color of solid compounds: shine orange-yellow; yields ≈ 60-85%; RF value in system 1 = 0.38-0.43. These intermediates were directly hydrolyzed to the correspondent acids 3,7,11.

*Nitro acid derivatives 3a-g, 7a, 11a-c, General procedure for synthesis of nitro acid derivatives*

The resulting esters 2a-g, 6s, 10a-c were dissolved in a mixture of absolute ethanol and 12 N HCl (3:7) under reflux at 80-90^°^C for 24-48 hrs. Then, the reaction was monitored by TLC until completion. At the end of the reaction, the mixture is poured on crushed ice to precipitate a pure product that was collected by filtration and left to dry at R.T. yields 80-90%.

- 1-cyclopropyl-6-fluoro-7-(2-methoxy-phenylamino)-8-nitro-4-oxo-1, 4-dihydro-quinoline-3-carboxylic acid (3a) (Code: 2-AnisCA).

- 1-cyclopropyl-6-fluoro-7-(3-methoxy-phenylamino)-8-nitro-4-oxo-1, 4-dihydro-quinoline-3-carboxylic acid (3b) (Code: 3-AnisCA).

- 1-cyclopropyl-6-fluoro-7-(4-methoxy-phenylamino)-8-nitro-4-oxo-1, 4-dihydro-quinoline-3-carboxylic acid (3c) (Code: 4-Anis CA).

- Synthesis of 1-cyclopropyl-6-fluoro-7-(4-ethyl-phenylamino)-8-nitro-4-oxo-1, 4-dihydro-quinoline-3-carboxylic acid (3d) (Code: 4-EtACA).

- Synthesis of 1-cyclopropyl-6-fluoro-7-(4-butyl-phenylamino)-8-nitro-4-oxo-1, 4-dihydro-quinoline-3-carboxylic acid (3e) (Code: 4-BuACA).

- Synthesis of 1-cyclopropyl-6-fluoro-7-(4-hexyl-phenylamino)-8-nitro-4-oxo-1, 4-dihydro-quinoline-3-carboxylic acid (3f) (Code: 4-HxACA).

- 1-cyclopropyl-7-(2, 4-dimethoxy-phenylamino)-6-fluoro-8-nitro-4-oxo-1, 4-dihydro-quinoline-3-carboxylic acid (3g) (Code: 2, 4-DMeOACA).

- 7-cyclohexylamino-1-cyclopropyl-6-fluoro-8-nitro-4-oxo-1, 4-dihydro-quinoline-3-carboxylic acid (7a) (Code: ChxCA).

- 1-ethyl-6-fluoro-7-(2-methoxy-phenylamino)-8-nitro-4-oxo-1, 4-dihydro-quinoline-3 carboxylic acid (11a) (Code: 2-AnisCEtA).

- 1-ethyl-6-fluoro-7-(3-methoxy-phenylamino)-8-nitro-4-oxo-1, 4-dihydro-quinoline-3-carboxylic acid (11b) (Code: 3-AnisCEtA).

- 7-(2, 4-dimethoxy-phenylamino)-1-ethyl-6-fluoro-8-nitro-4-oxo-1, 4-dihydro-quinoline-3-carboxylic acid (11c) (Code: 2, 4-DMeOACEtA).

*Synthesis of reduced derivatives 4a-g, 8a, 12a-c*

*General procedure for reduced series*

A mixture of the acids 3a-g, 7a, 11a-c (1-ethyl or cycloprpyl-6-fluoro- 7-(substituted aniline) -8-nitro-4-oxo-1,4- dihydroquinoline-3-carboxylic acid (3a-g, 7a, 11a-c ) in 15 mL of 12N HCl, was left stirring in ice bath (2-5°C) for 20 min. Then, the ice bath was removed and 5 molar excess of stannous chloride (SnCl_2_) was added portion wise and the reaction mixture left stirring overnight and monitored by TLC until completion. Then, the reaction mixture was poured on crushed ice to precipitate product that is collected by filtration and left to dry. Target compounds were obtained in good yields with yellowish green color.

- 8-amino-1-cyclopropyl-6-fluoro-7-(2-methoxy-phenylamino)-4-oxo-1, 4-dihydro-quinoline-3-carboxylic acid (4a) (Code: R-2-AnisCA).

- 8-amino-1-cyclopropyl-6-fluoro-7-(3-methoxy-phenylamino)-4-oxo-1, 4-dihydro-quinoline-3-carboxylic acid (4b) (Code: R-3-AnisCA).

- 8-amino-1-cyclopropyl-6-fluoro-7-(4-methoxy-phenylamino)-4-oxo-1, 4-dihydro-quinoline-3-carboxylic acid (4c) (Code: R-4-AnisCA).

- 8-Amino-1-cyclopropyl-6-fluoro-7-(4-ethyl-phenylamino)-4-oxo-1, 4-dihydro-quinoline-3-carboxylic acid (4d) (Code: R-4-EtACA).

- 8-Amino-1-cyclopropyl-6-fluoro-7-(4-butyl-phenylamino)-4-oxo-1, 4-dihydro-quinoline-3-carboxylic acid (4e) (Code: R-4-BuACA).

- 8-Amino-1-cyclopropyl-6-fluoro-7-(4-hexyl-phenylamino)-4-oxo-1, 4-dihydro-quinoline-3-carboxylic acid (4f) (Code: R-4-HxACA).

- 8-amino-1-cyclopropyl-7-(2, 4-dimethoxy-phenylamino)-6-fluoro-4-oxo-1, 4-dihydro-quinoline-3-carboxylic acid (4g) (Code: R-2, 4-DMeOACA).

- Synthesis of 8-amino-7-cyclohexylamino-1-cyclopropyl-6-fluoro-4-oxo-1, 4-dihydro-quinoline-3-carboxylic acid (8a) (Code: R-CHxCA).

- 8-amino-1-ethyl-6-fluoro-7-(2-methoxy-phenylamino)-4-oxo-1, 4-dihydro-quinoline-3-carboxylic acid (12a) (Code: R-2-AnisCEtA).

- 8-amino-1-ethyl-6-fluoro-7-(3-methoxy-phenylamino)-4-oxo-1, 4-dihydro-quinoline-3-carboxylic acid (12b) (Code: R-3-AnisCEtA).

- 8-amino-7-(2, 4-dimethoxy-phenylamino)-1-ethyl-6-fluoro-4-oxo-1, 4-dihydro-quinoline-3-carboxylic acid (12c) (Code: R-2, 4-DMeOCEtA).

*Synthesis of Triazolo- derivatives 5a-g, 9a, 13a-c.*

*General procedure to prepare Triazolo- derivatives*

A mixture of the reduced compounds 4a-g, 8a, 12a-c (8-amino-1-ethyl/cyclopropyl)-6-fluoro-7-(substituted aniline) -4-oxo-1,4- dihydroquinoline-3-carboxylic in 20 mL of aqueous HCl, was stirred in ice bath (2-5°C) for 15 minutes. Sodium nitrite solution (NaNO2) dissolved in 10 mL H2O was added drop wise. The reaction mixture was left stirring 24 hr. The reaction progress was monitored by TLC until no starting material left. Then, the reaction mixture was poured on crushed ice to precipitate product that is collected by filtration and left to dry. Triazolo targets were produced with good yields.

- 9-cyclopropyl-4-fluoro-3-(2-methoxy-phenyl)-6-oxo-6, 9-dihydro-3H-[1, 2, and 3] triazolo [4, 5-h] quinoline-7-carboxylic acid (5a) (Code: T-2-AnisCA).

- 9-cyclopropyl-4-fluoro-3-(3-methoxy-phenyl)-6-oxo-6, 9-dihydro-3H-[1, 2, and 3] triazolo [4, 5-h] quinoline-7-carboxylic acid (5b) (Code: T-3-AnisCA).

- 9-cyclopropyl-4-fluoro-3-(4-methoxy-phenyl)-6-oxo-6, 9-dihydro-3H-[1, 2, and 3] triazolo [4, 5-h] quinoline-7-carboxylic acid (5c) (Code: T-4-AnisCA).

- 9-cyclopropyl-4-fluoro-3-(4-ethyl-phenyl)-6-oxo-6,9-dihydro-3H-[1,2,3] triazolo[4,5-h]quinoline-7-carboxylic acid (5d) (Code: T-4-EtACA).

- 9-cyclopropyl-4-fluoro-3-(4-butyl-phenyl)-6-oxo-6,9-dihydro-3H-[1,2,3] triazolo[4,5-h]quinoline-7-carboxylic acid (5e) (Code: T-4-BuACA).

- 9-cyclopropyl-4-fluoro-3-(4-hexyl-phenyl)-6-oxo-6,9-dihydro-3H-[1,2,3] triazolo[4,5-h]quinoline-7-carboxylic acid (5f) (Code: T-4-HxACA).

- 9-cyclopropyl-3-(2,4-dimethoxy-phenyl)-4-fluoro-6-oxo-6,9-dihydro-3H-[1,2,3] triazolo[4,5-h]quinoline-7-carboxylic acid (5g) (Code: T-2,4DMeOACA).

- 3-cyclohexyl-9-cyclopropyl-4-fluoro-6-oxo-6, 9-dihydro-3H-[1, 2, and 3] triazolo [4, 5-h] quinoline-7-carboxylic acid (9a) (Code: T-CHxCA).

- 9-ethyl-4-fluoro-3-(2-methoxy-phenyl)-6-oxo-6, 9-dihydro-3H-[1, 2, and 3] triazolo [4, 5-h] quinoline-7-carboxylic acid (13a) (Code: T-2-AnisCEtA).

- 9-ethyl-4-fluoro-3-(3-methoxy-phenyl)-6-oxo-6, 9-dihydro-3H-[1, 2, and 3] triazolo [4, 5-h] quinoline-7-carboxylic acid (13b) (Code: T-3-AnisCEtA).

- 3-(2,4-dimethoxy-phenyl)-9-ethyl-4-fluoro-6-oxo-6,9-dihydro-3H-[1,2,3] triazolo[4,5-h]quinoline-7-carboxylic acid (13c) (Code: T-2,4-DMeOACEtA).

*In vitro cell viability assay*

The reference antineoplastic agent used for anti-proliferative assay cisplatin was procured from Sigma (St. Luis, MO, USA). The cytotoxicity measurements were determined using Sulforhodamine B (SRB) colorimetric assay for cytotoxicity screening. The mechanism of reduction of cell viability was adopted as described previously (Arabiyat et al., 2016a,b; 2017; Alabsi et al., 2018; Jumah, 2018; AlShahrabi et al., 2020). Human periodontal ligament fibroblasts (PDL) are a primary normal cell culture for verification of selective cytotoxicity. As the robust and classical antineoplastic reference agent, cisplatin (1-100 μg/mL) was recruited for comparison purposes (Arabiyat et al., 2016a,b; 2017; Alabsi et al., 2018; Jumah, 2018; AlShahrabi et al., 2020). Triplicate assay approach was performed and the calculated anti-proliferative activities were reported as IC_50_ mean values ± SD (n=3). Selectivity ratio as indicative factor of the safety of compounds was calculated by dividing IC_50_ of tested compound on normal fibroblasts by IC_50_ of the same compound on specific pathological cell line (Hoffman et al., 2011).

*Statistical analysis*

Values are presented as mean ± SD (standard deviation) of 3 independent experiments. Statistical differences between reference agent and different treatment compounds were determined using Graph Pad Prism software unpaired t-test (version 5.01 for windows; Graph Pad software, San Diego, CA, USA). Values were considered significantly different if P< 0.05 and highly significantly different if P<0.01 and P<0.001.

## Results

*Chemistry*

*Targeted compounds scheme*

Target compounds (Arabiyat et al., 2016a,b; 2017; Alabsi et al., 2018; Jumah, 2018; AlShahrabi et al., 2020) were prepared as anti-pancreatic lipase and anticancer activities. Same compounds were rescreened and evaluated for their dual antiglycation-antiinflamation action (Hamdan et al., 2019; AL-AShahrabi et al., 2020) 

*Growth inhibition activity of studied compounds in the eight cell lines*

Antiproliferative effectiveness of tested FQs derivatives against eight cell lines (**A549, A375.S2, MCF7, HELA, K562, PANC 1, T47D**) and **PDL** fibroblasts was demonstrated with respective IC_50_ values (Table 1). Each cell line showed a distinctive response profile to the set of **36** tested compounds. Cisplatin’s antiproliferative efficacies in all cell lines were further illustrated for potency comparisons. Table 1 showed each FQ’s selective cytotoxicity index. Table 1 revealed that our **36** compounds could demonstrate moderate to substantial activity yet still few compounds were of incomparably less appreciable activity. 

Table 1 illustrates numerous effective FQs derivatives with greater potency than cisplatin on the same panel of cancer cell lines. Table 1 highlights the 40% of FQ compounds with leas reasonably appreciable selectivity index and lack of differential cytotoxicity like cisplatin’s. Interestingly compound **4**c had significantly higher cytotoxicity than cisplatin (P value < 0.001) on A549 lung carcinoma cell line. While 7a exhibited exceptional cytotoxicity in Nano molarity IC_50_ on Hela cervical adenocarcinoma; the rest exerted less appreciable antiproliferative effectiveness in malignant melanoma A375.S2 (Table 1) 

Similarly compounds **4f**, **13e** and** 1A** exhibited cytotoxicity (P value <0.0001) on MCF7 cell line and 4f on PANC 1 cancer cell line (P value <0.0001) that were of comparable potencies vs. cisplatin. On the other hand **4e** and **5a** compounds were equipotent to cisplatin on T47D; **5b** (P value <0.001) **11a **(P value <0.001) proved more potent than cisplatin on T47D (P value <0.0001). **12c** and **7a** were alike in cisplatin’s potency on the chronic myelogenous leukaemia K562; Effective FQ derivatives **3e, 4e, 3b, 5b, 11a, 12a** and **13a** (P <0.05 and <0.001) were all pronouncedly more potent than cisplatin on the same K562 (Table 1).

*Antimicrobial activity of studied compounds on Gram positive or negative bacteria or yeast*

*Qualitative assessment for antimicrobial activity*

Supplementaries 1, 2 and 3 showed the tested compounds with potential antimicrobial activity against *Staphylococcus aureus* (*S. aureus*) *ATCC 6538*, *Escherichia coli *(*E. coli*) *ATCC 25922* and* Candida albican*s (*C. albicans*) *ATCC 1023*. Each compound had different zones of inhibition. As for *S. aureus* only two out of 36 compounds did not have zone of inhibition. Sixteen out of 36 compounds did not have zones of *E. coli* inhibition and seven out of 36 compounds lacked zone of yeast inhibition.

*Quantitative assessment for antimicrobial activity*

Table 4 supplementary shows the MIC values of the tested compounds against S. aureus. Compounds 4e, 4g, 5a, 5d, and 13c were significantly more potent than ciprofloxacin (P value <0.001). Compound 4b was comparably equipotent as ciprofloxacin (P>0.05). Table 5 supplementary showed the MIC values of the tested compounds against E. coli. Incomparably, tested FQs were less potent vs. ciprofloxacin with a broad range of measurable efficacies. Table 6 supplementary shows the antimycotic MIC values of the tested compounds against *C. albicans*. Distinctively only three (**4e**, **5e** and **8e**) out of 36 FQs were more potent than antimycotic flucanozole (P <0.001). The rest proved inactive.

**Figure 2:**
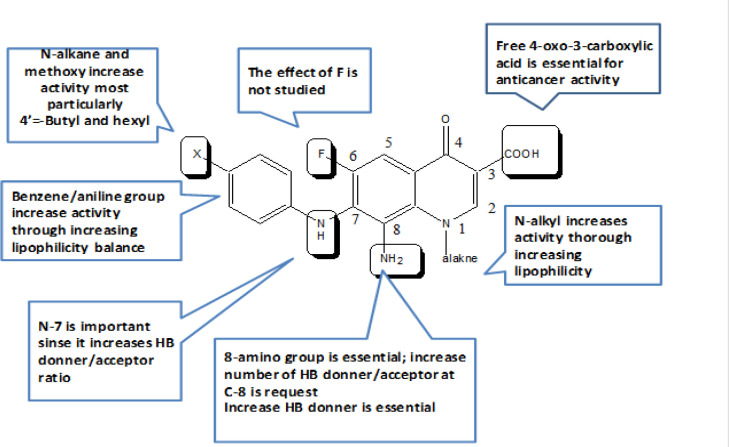
SAR Requirements for of Functionalities in Relevance of Growth Inhibition Activity of FQs Class

*Structure activity relationship of antiproliferative activity (SARS;* Figure 2 *)*

Tables 1 and 2 specify low or negligible anti-proliferative activities for the ethyl esters **1E** and **2h** compared to the free acid **1A**; indicating the need for free 4-Oxo-3-COOH acidic ionisable groups. The free acidic COOH group possibly contributes to their anticancer activity through ionic bonds. It is well documented and proved by our previous work that the 4-Oxo-3-COOH contributes to any activity through chelation with di- and trivalent metals. (Kasabri et al., 2014; Arabiyat et al., 2016a,b; Kasabri et al., 2017; Alabsi et al., 2018; Jumah 2018; Abdul Fattah et al., 2019; Arabiyat et al., 2020). In addition, it provides extra number of hydrogen bond donor and acceptor mediate receptors inter action. This result has led us to exclude ester derivatives 2 from screening and focus on acids 3-13.

 It was apparent that C-7 aniline lipophilic group has increased activity due to lipophilicity. The acids with C-7 anilines (**4b**, **4c**, **4f**, **4e**, **7a**, **12a**, and **12c**) are the only compounds which showed nanomolar IC_50_ values; whereas compound 1A which lack this lipophilic group have much lower activity. It was evident that the strong reduced series (**4b**,** 4c**, **4f**, **4e**, **12a**, **12c**) have shown the best anti-proliferative activity, followed by nitro group then triazolo, against most cancer cells. This shows that the C-8 amino group (NH_2_) has an essential role in activity possibly through hydrogen bonding with the receptor. It is well-known that any free amino group increases the number of H-B acceptor: donor ratio and for sure increases the chances for better fit of the compound with its receptors. NH_2_ increase such ratio to 2:1 which is significant to make 3 H-B.

**Table 1:** Cytotoxicity in vitro (as of %Control) IC_50_ Value in µM (µg/mL) in a Diverse Panel of Cancer Cell Lines vs. cisplatin . Selectivity index (SI) of the tested compounds against PDL Fibroblasts vs. cisplatin’s

Treatment	A549 cell line		A375.S2 cell line			MCF7 cell line				HELA cell line			K562 cell line		PANC 1 cell line				T47D cell line			IC_50_ value PDL Fibroblasts µM (µg/mL) SI	
Nitro series FQs (3, 7,11)																							
3a 2-Anis CA	96.77± 8.75 *** (8.84 ± 3.51)		1024.21 ± 19.82*** (411.07 ± 48.09)			67.78 ± 12.77** (27.20 ± 5.12)				103.24 ± 7.66*** (41.44 ± 3.07)			1.62 ± 0.16*** (0.71 ± 0.07)		187.44 ± 2.87*** (75.23 ± 1.15)				NI			183.97 ± 3.69*** (76.05 ± 1.52) SI 2.71 (MCF7)	
3b 3-AnisCA	NI		NI			NI				NI			NI		NI				NI**			NI SI NI	
3c 4-Anis CA	64.41 ± 4.82*** (26.62 ± 1.99)		274.14 ± 46.59*** (113.32 ± 19.26)			54.17 ± 7.12*** (22.39 ± 2.95)				197.58 ± 11.12*** (81.67 ± 4.60)			79.08 ± 8.69*** (32.69 ± 3.59)		40.72 ± 0.36* (16.83 ± 0.15)				66.01 ± 9.15*** (27.29 ± 3.78)			107.15 ± 5.61** (44.29 ± 2.32) SI 2.63 (PANC 1)	
3d 4-EtACA	NI		372.35 ± 26.30*** (153.18 ± 10.82)			NI				NI			NI		NI				NI			728.35 ± 118.74*** (299.63 ± 48.85) SI 1.95 (A375.S2)	
3e 4-BuACA	64.19 ± 5.96*** (28.21 ± 2.62)		144.44 ± 23.45*** (63.47 ± 10.31)			428.49 ± 18.38*** (188.37 ± 8.15)				1133.14 ± 65.57*** (497.95 ±72.76)			1.62 ± 0.16*** (0.71 ± 0.07)		12.24 ± 1.81* (5.38 ± 0.79)				405.72 ± 32.27*** (178.29 ± 4.18)			711.19 ± 44.94*** (312.52 ± 19.75) SI 439.01 (K562)	
3f 4-HxACA	63.94 ± 3.69*** (29.89 ± 1.73)		63.13 ± 4.51*** (29.51 ± 2.11)			135.44 ± 2.49*** (63.32 ± 1.16)				7.44 ± 0.78*** (3.48 ± 0.36)			64.12 ± 4.78*** (29.98 ± 2.23)		1667.22 ±208.57*** (779.41 ± 97.50)				141.19 ± 12.69*** (66.01 ± 5.93)			119.11 ± 19.69*** (55.68 ± 9.20) SI 16.01 (HELA)	
3g 2,4-DMeOACA	NI		NI			NI				NI			NI		NI				NI			NI SI NI	
7a CHxCA	240.32 ± 3.76*** (93.58 ± 1.47)		246.15 ± 43.85*** (101.75 ± 18.13)			NI				0.40 ± 0.05** (0.16 ± 0.02)			28.23 ± 2.04ns 10.99 ± 0.79		13.23 ± 1.40*** (5.15 ± 0.55)				51.24 ± 7.27ns (19.95 ± 2.83)			29.87 ± 3.56*** (11.63 ± 1.39) SI 74.66 (HELA)	
11a 2-AnisCEtA	96.77 ± 8.75*** (8.84 ± 3.51)		1024.21 ± 19.82*** (411.07 ± 48.09)			67.78 ± 12.77** (27.20 ± 5.12)				103.24 ± 7.66*** (41.44 ± 3.07)			15.89 ± 1.75* (6.38 ± 0.70)		187.44 ± 2.87*** (75.23 ± 1.15)				22.82 ± 2.03** (9.16 ± 0.82)			156.23 ± 11.71*** (62.70 ± 4.70) SI 9.83 (K562)	
11b 3-AnisCEtA	NI		NI			NI				196.85 ± 38.34*** (79.01 ± 15.39)			58.02 ± 9.12** (23.29 ± 3.66)		NI				NI			NI SI NI	
11c 2,4- DMeOACEtA	NI		NI			NI				NI			NI		123.54 ± 7.80*** (53.29 ± 3.37)				430.96 ± 43.69*** (185.90 ± 18.84)			285.49 ± 28.73*** (123.15 ± 12.40) SI 2.31 (PANC 1)	
Reduced series FQs (4, 8, 12)																							
4a R-2-AnisCA	153.07 ± 3.20*** (58.73 ± 1.23)		72.34 ± 7.45*** (27.76 ± 2.86)			NI				40.38 ± 6.03*** (15.49 ± 2.31)			44.01 ± 3.88*** (16.87 ± 1.49)		113.08 ± 3.40** (43.39 ± 1.31)				95.84 ± 1.65*** (36.77 ± 0.63)			140.07 ±10.54** (53.75 ± 4.04) SI 3.46 (HELA)	
4b R-3-AnisCA	228.55 ± 22.37*** (87.62 ± 8.58)		119.88 ± 6.90*** (45.96 ± 2.64)			176.59 ± 4.29*** (67.7 ± 1.64)				73.36 ± 1.52** (28.12 ± 0.58)			16.96 ± 2.57*** (6.50 ± 0.99)		154.53 ± 7.48*** (59.24 ± 2.87)				149.30 ± 3.08*** (57.24 ± 1.18)			275.63 ± 20.15*** (105.67 ± 7.73) SI 16.25 (K562)	
Treatment	A549 cell line		A375.S2 cell line			MCF7 cell line				HELA cell line			K562 cell line		PANC 1 cell line				T47D cell line			IC50 value PDL Fibroblasts µM (µg/mL) SI	
Reduced series FQs (4, 8, 12)																						
4c R-4-Anis CA		5.11 ± 0.77*** (1.96 ± 0.29)			247.78 ± 9.28*** (94.99 ± 3.56)			57.36 ± 6.93*** (21.99 ± 2.66)	255.00 ± 2.15** (97.76 ± 0.82)			52.10 ± 6.90*** (19.97 ± 2.65)			72.10 ± 2.09* (27.64 ± 0.80)		77.20 ± 4.45*** (29.60 ± 1.70)				138.36 ± 8.26*** 53.04 ± 3.17 SI 27.07 (A562)	
4d R-4-EtACA		20.24 ± 2.86* (7.72 ± 1.09)			47.05 ± 2.39*** (17.94 ± 0.91)			21.34 ± 2.39** (8.14 ± 0.91)	135.58 ± 4.58*** (51.71 ± 1.75)			41.79 ± 4.98* (15.94 ± 1.90)			65.312 ± 6.48*** (24.91 ± 2.47)		53.68 ± 7.48ns (20.47 ± 2.85)				50.38 ± 4.28*** (19.21 ± 1.63) SI 2.49 (A562)	
4e R-4-BuACA		25.1 ± 3.52*** (10.30 ± 1.44)			79.22 ±7.11*** (32.44 ± 2.91)			107.40 ± 3.26*** (43.97 ± 1.33)	67.30 ± 6.64*** (27.56 ± 2.72)			0.005 ± 0.0009*** (0.002 ±0.0004)			20.76 ± 0.35* (8.5 ± 0.14)		44.07 ± 1.36ns (18.04 ± 0.56)				42.96 ± 3.02*** (18.88 ± 1.33) SI 8592 (K592)	
4f R-4-HxACA		204.45 ± 29.34*** (89.45 ±12.84)			244.06 ± 12.20*** (106.78 ± 5.34)			0.30 ± 0.03*** (0.13 ± 0.01)	30.66 ± 3.79*** (13.41 ± 1.66)			69.42 ± 6.64** (30.37 ± 2.90)			0.11 ± 0.02*** (0.05 ± 0.01)		307.72 ± 27.43*** (134.63 ± 12.00)				351.94 ± 7.36*** (153.98 ± 3.22) SI 3199.45 (PANC 1)	
4g R 2,4-DMeOACA		708.61 ± 48.55*** (305.69 ±20.94)			806.53 ± 27.10*** (347.94 ± 11.69)			316.65 ± 35.21*** (136.60 ±15.19)	277.02 ± 38.98*** (119.51 ±16.82)			NI			309.89 ± 8.02*** (128.11 ± 3.31)		1008.00 ± 7.11*** (434.85 ± 3.07)				NI SI NI	
8a R-CHxCA		43.97 ± 1.93*** (15.80 ± 0.69)			3.69 ± 0.69*** (1.33 ± 0.25)			NI	125.23 ± 21.85*** (45.01 ± 7.85)			67.91 ± 6.76** (24.41 ± 2.43)			136.63 ± 3.70** (49.10 ± 1.33)		148.06 ± 19.43** (53.21 ± 6.98)				49.44 ± 2.71*** (17.77 ± 0.98) SI 12.48 (A375.S2)	
12a R-2-AnisCETA		NI			372.59 ± 71.91*** (138.37 ± 26.71)			125.32 ± 18.29*** (46.54 ± 6.79)	25.45 ± 3.92*** (9.45 ± 1.46)			20.99 ± 0.62* (7.79 ± 0.23)			141.68 ± 9.31*** (52.61 ± 3.46)		83.54 ± 3.05** (31.02 ± 1.13)				170.10 ± 15.09*** (63.17 ± 5.60) SI 8.10 (K562)	
12b R-3-AnisCEtA		162.62 ± 24.64*** (60.39 ± 9.15)			112.29 ± 1.01*** (41.70 ± 0.38)			491.55 ± 7.28*** (182.54 ± 2.70)	225.07 ± 33.99*** (83.58 ± 12.62)			35.41 ± 3.80ns (13.15 ± 1.41)			35.41 ± 3.80*** (13.15 ± 1.41)		168.89 ± 11.87*** (62.72 ± 4.41)				149.59 ± 6.73** (55.55 ± 2.50) SI 4.22 (K562)	
12c R-2,4-DMeOACEtA		40.94 ± 1.37*** (16.43 ± 0.55)			67.35 ± 5.11*** (27.03 ± 2.05)			100.67 ± 16.40*** (40.41 ± 6.58)	38.84 ± 1.91*** (15.59 ± 0.77)			26.44 ± 4.54ns (10.61 ± 1.82)			93.50 ± 2.38*** (37.53 ± 0.95)		430.96 ± 43.69ns (185.90 ± 18.84)				43.75 ± 6.21*** (17.56 ± 2.49) SI 1.65 (K562)	
Triazolo series FQs (5, 9, 13)																						
5a T-2-AnisCA		NI			170.97 ± 12.80*** (67.25 ± 5.04)			44.28 ± 2.36*** (17.42 ± 0.92)	299.04 ± 11.43*** (123.61 ± 4.72)			41.67 ± 6.86** (16.39 ± 2.70)			200.58 ± 18.73*** (78.90 ± 7.37)		42.07 ± 6.17ns (16.55 ± 2.43)				279.49 ± 27.41** (109.94 ± 10.78) SI 6.71 (K562)	
5b T-3-AnisCA		196.38 ± 17.30*** (77.25 ± 6.80)			363.58 ± 64.76** (143.02 ± 25.48)			112.39 ± 4.07*** (44.21 ± 1.60)	66.88 ± 2.73*** (26.31 ± 1.08)			11.60 ± 0.61*** (4.56 ± 0.24)			178.39 ± 14.94** (70.17 ± 5.88)		17.99 ± 2.74*** (7.08 ± 1.08)				143.27 ± 10.79*** (56.36 ± 4.24) SI 12.35 (K562)	
5c T-4-AnisCA		427.45 ± 22.96*** (168.15 ± 9.03)			453.46 ± 19.04*** (178.38 ± 7.49)			NI	453.97 ± 19.11*** (178.58 ± 7.52)			247.63 ± 44.57*** (97.41 ± 17.53)			NI		397.56 ± 15.85*** (156.39 ± 6.23)				496.12 ± 52.46*** (195.16 ± 20.64) SI 2.00 (K562)	
5d T-4-EtACA		161.03 ± 1.28*** (63.03 ± 0.50)			123.85 ± 14.15** (48.48 ± 5.54)			176.65 ± 8.20*** (69.14 ± 3.21)	1142.14 ± 82.42*** (447.03± 71.40)			162.88 ± 22.58*** (63.75 ± 8.84)			162.97 ± 7.66*** 63.77 ± 2.997		371.79 ± 10.67*** (145.52 ± 4.18)				164.76 ± 16.13*** (64.49 ± 6.31) SI 1.33 (A375.S2)	
Triazolo series FQs (5, 9, 13)																						
5e T-4-BuACA		181.21 ± 9.46** (76.01 ± 3.97)		102.77 ± 3.85*** (43.11 ± 1.61)			105.30 ± 6.18*** (44.16 ± 2.59)				363.69 ± 22.76** (152.55 ± 9.55)			49.04 ± 7.20** (20.57 ± 3.02)		87.11 ± 0.53* (36.54 ± 0.223)		97.63 ± 2.43*** (40.95 ± 1.02)		72.58 ± 3.56*** (30.44 ± 1.49) SI 1.48 (K562)		
5f T-4-HxACA		361.98 ± 18.39*** (161.99 ± 8.23)		508.36 ± 53.18** (227.49 ± 23.80)			181.13 ± 23.13*** (81.06 ± 10.35)				3.59 ± 0.05*** (1.61 ± 0.02			31.50 ± 2.92*** (14.10 ± 1.31)		119.37 ± 3.67** (53.42 ± 1.64)		231.66 ± 14.33*** (103.67 ± 6.41)		474.44 ± 33.52*** (212.31 ± 15.00) SI 132.15 (HELA)		
5g T-2,4 DMeOACA		473.83 ± 21.25*** (204.87 ± 9.00)		271.90 ± 8.86*** (117.56 ± 3.75)			143.21 ± 27.34*** (61.923 ±11.57)				165.94 ± 25.96*** (71.75 ± 10.99)			55.98 ± 8.04*** (24.20 ± 3.40)		NI		236.54 ± 13.50*** (102.27 ± 5.72)		236.54 ± 13.50** (102.27 ± 5.72) SI 4.23 (K562)		
9a T-CHxCA		222.75 ± 14.11** (82.28 ± 5.21)		215.42 ± 3.29*** (79.57 ± 1.22)			133.30 ± 14.24*** (49.24 ± 5.26)				74.83 ± 1.67** (27.64 ± 0.62)			NI		162.45 ± 6.78** (60.007 ± 2.50)		298.93 ± 3.48*** (110.42 ± 1.29)		252.14 ± 11.63*** (93.14 ± 4.29) SI 3.36 (HELA)		
13a T-2-AnisCEtA		247.33 ± 3.78*** (94.32 ± 1.44)		534.60 ± 12.58** (203.87 ± 4.80)			NI				52.60 ± 6.86*** (20.06 ± 2.62)			6.98 ± 1.28 (2.66 ± 0.49)		310.21 ± 17.58** 118.30 ± 6.71		57.51 ± 2.27*** (21.93 ± 0.87)		275.66 ± 20.73*** (105.13 ± 7.90) SI 40.01 (K562)		
13b T-3-AnisCEtA		251.39 ± 24.13*** (95.87 ± 9.20)		102.15 ± 8.88*** (38.96 ±3.39)			417.40 ± 22.75** (159.18 ± 8.68)				41.93 ± 3.55*** (15.99 ± 1.35)			58.03 ± 2.22** (22.13 ± 0.85)		NI		117.15 ± 9.15*** (44.68 ± 3.49)		331.83 ± 14.63*** (126.55 ± 5.58) SI 7.19 (HELA)		
13c T-2,4-DMeOACEt A		662.15 ± 70.75*** (272.40 ±29.10)		487.42 ± 73.83*** (200.51 ± 30.37)			4.15 ± 0.77** (1.71 ± 0.32)				434.84 ± 71.73*** (178.89 ±29.51)			68.66 ± 8.04*** (28.25 ± 3.31)		137.63 ± 9.97** (56.62 ± 4.10)		181.55 ± 1.04*** (74.69 ± 0.43)		171.61 ± 17.76*** (70.60 ± 7.31) SI 41.35 (MCF7)		
1A CIPRO ACID “CA”		57.88 ± 1.75*** (20.36 ± 0.62)		10.01 ± 1.22** (3.52 ± 0.43)			3.319 ± 0.59*** (1.167 ± 0.208)				19.78 ± 1.48*** (7.02 ± 0.53)			NI		59.15 ± 10.55*** (20.98 ± 3.74)		NI		186.20 ± 12.08*** (60.82 ± 3.95) SI 2.35 (PANC1)		
1E CIPRO ESTER “CE”		447.30 ± 45.71*** (200.17 ± 20.46)		271.03 ± 17.95** (121.29 ± 8.03)			220.12 ± 20.18*** (71.90 ± 6.59)				132.57 ± 4.78*** (43.31 ± 1.56)			99.11 ± 7.50*** (32.38 ± 2.45)		79.29 ± 6.53*** (25.90 ± 2.13)		108.40 ± 11.35** (35.41 ± 3.71)		6.75 ± 0.88 ** (2.37 ±0.31) SI 2.03 (MCF7)		
2H PF ANILIN “CE		447.30 ± 45.71*** (200.17 ±20.46)		271.03 ± 17.95*** (121.29 ± 8.03)			NI				1507.09 ± 68.18*** (647.10 ±29.27)			805.72 ± 59.18** (345.95 ±25.41)		485.19 ± 29.31*** (208.33 ± 12.59)		833.08 ± 59.20*** (344.36 ± 24.47)		559.94 ± 87.48 *** (231.46 ± 36.16) SI 2.07 (A375.S2)		
Cisplatin		12.27 ± 2.05 (3.68 ± 0.62)		0.7 ± 0.1 (0.22 ± 0.03)			11.62 ± 1.06 (3.49 ± 0.32)				0.18 ± 0.03 (0.055 ± 0.008)			29.3 ± 5 (8.8 ± 1.5)		7.01 ± 1.17 (2.10 ± 0.35)		45.15 ± 7.84 (13.55 ± 2.35)		0.71 ± 0.13 (0.21 ± 0.04) SI 3.94 (HELA)		

Results are mean ± SD (n = 3-4 independent replicates). IC50 values (concentration at which 50% inhibition of cell proliferation took place in comparison to non-induced basal 72 h incubations) were calculated within 0.1-200 μg/mL range. NI is lack of cytotoxicity within the tested 0.1-200 μg/mL concentration range. P-value calculated by unpaired t-test between test compound IC50 values and cisplatin's (μM) using Graph Pad Prism software version 5.0.1.* when P<0.05 and ** when P< 0.01 or 0.001, *** when P<0.0001, NS: not significantly different from reference agent; Treat, treatment.

**Table 2:** Cytotoxicity (as of % Control) IC_50_ Values of 50 and Below of 50 µM

Code	Treatment	A549 cell line	A375.S2 cell line	MCF7 cell line	HELA cell line	K562 cell line	PANC 1 cell line	T47D cell line
Nitro series FQs (3, 7,11)								
3a	2-Anis CA					1.62 ± 0.16*** (0.71 ± 0.07)		
3c	4-Anis CA					40.72 ± 0.36* (16.83 ± 0.15)		
3e	4-BuACA					1.62 ± 0.16*** (0.71 ± 0.07)		
3f	4-HxACA				7.44 ± 0.78*** (3.48 ± 0.36)			
7a	CHxCA				0.40 ± 0.05** (0.16 ± 0.02)	28.23 ± 2.04^ns^ 10.99 ± 0.79	13.23 ± 1.40*** (5.15 ± 0.55)	
11a	2-AnisCEtA					15.89 ± 1.75* (6.38 ± 0.70)		22.82 ± 2.03** (9.16 ± 0.82)
Reduced series FQs (4, 8, 12)								
4a	R-2-AnisCA				40.38 ± 6.03*** (15.49 ± 2.31)	44.01 ± 3.88*** (16.87 ± 1.49)		
4b	R-3-AnisCA				16.96 ± 2.57*** (6.50 ± 0.99)			
4c	R-4-Anis CA	5.11 ± 0.77*** (1.96 ± 0.29)						
4d	R-4-EtACA	20.24 ± 2.86* (7.72 ± 1.09)	47.05 ± 2.39*** (17.94 ± 0.91)	21.34 ± 2.39** (8.14 ± 0.91)		41.79 ± 4.98* (15.94 ± 1.90)		
4e	R-4-BuACA	25.1 ± 3.52*** (10.30 ± 1.44)				0.005 ± 0.0009*** (0.002 ±0.0004)	20.76 ± 0.35* (8.5 ± 0.14)	44.07 ± 1.36^ns^ (18.04 ± 0.56)
4f	R-4-HxACA			0.30 ± 0.03*** (0.13 ± 0.01)			0.11 ± 0.02*** (0.05 ± 0.01)	
8a	R-CHxCA	43.97 ± 1.93*** (15.80 ± 0.69)	3.69 ± 0.69*** (1.33 ± 0.25)					
12a	R-2-AnisCETA				25.45 ± 3.92*** (9.45 ± 1.46)	20.99 ± 0.62* (7.79 ± 0.23)		
12b	R-3-AnisCEtA					35.41 ± 3.80^ns^ (13.15 ± 1.41)	35.41 ± 3.80*** (13.15 ± 1.41)	
12c	R-2,4-DMeOACEtA	40.94 ± 1.37*** (16.43 ± 0.55)			38.84 ± 1.91*** (15.59 ± 0.77)	26.44 ± 4.54^ns^ (10.61 ± 1.82)		
Triazolo series FQs (5, 9, 13)								
5a	T-2-AnisCA			44.28 ± 2.36*** (17.42 ± 0.92)		41.67 ± 6.86** (16.39 ± 2.70)	NI	42.07 ± 6.17^ns^ (16.55 ± 2.43)
5b	T-3-AnisCA					11.60 ± 0.61*** (4.56 ± 0.24)		17.99 ± 2.74*** (7.08 ± 1.08)
5e	T-4-BuACA					49.04 ± 7.20** (20.57 ± 3.02)		
5f	T-4-HxACA				3.59 ± 0.05*** (1.61 ± 0.02)			
13b	T-3-AnisCEtA				41.93 ± 3.55*** (15.99 ± 1.35)			
13c	T-2,4-DMeOACEt A			4.15 ± 0.77** (1.71 ± 0.32)				
A2	CIPRO ESTER “CE”	10.01 ± 1.22** (3.52 ± 0.43)		3.319 ± 0.59*** (1.167 ± 0.208)	19.78 ± 1.48*** (7.02 ± 0.53)			
Cisplatin		12.27 ± 2.05 (3.68 ± 0.62)	0.7 ± 0.1 (0.22 ± 0.03)	11.62 ± 1.06 (3.49 ± 0.32)	0.18 ± 0.03 (0.055 ± 0.008)	29.3 ± 5 (8.8 ± 1.5)	7.01 ± 1.17 (2.10 ± 0.35)	45.15 ± 7.84 (13.55 ± 2.35)

NI, No inhibition zone; P values of *, **, *** vs. cisplatin, IC_50_ values (concentration at which 50% inhibition of cell proliferation took place in comparison to non-induced basal 72 h incubations) were calculated within 0.1-200 μg/mL range, PDL Periodontal Ligament Fibroblast. Breast cancer MCF7 and T47D cell lines; Leukaemia K562 cell lines; Pancreatic PANC1 cancer cell lines; Melanoma A375.S2 cancer cell lines; Lung cancer A569 cancer cell lines and Cervical cancer HELA cell lines.

**Figure 3:**
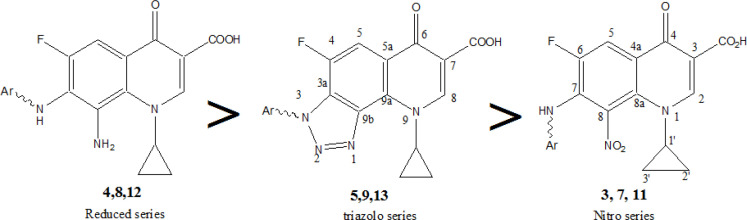
Order of Growth Inhibition Activity Based on Optimized Structural Classification of Tested Compounds in Strong Group (Table 3)

**Scheme 1:** Targeted Compounds 2-5 (a-f) (Arabiyat et al. 2016a,b; 2017; Alabsi et al. 2018; Jumah 2018; AlShahrabi et al., 2020)

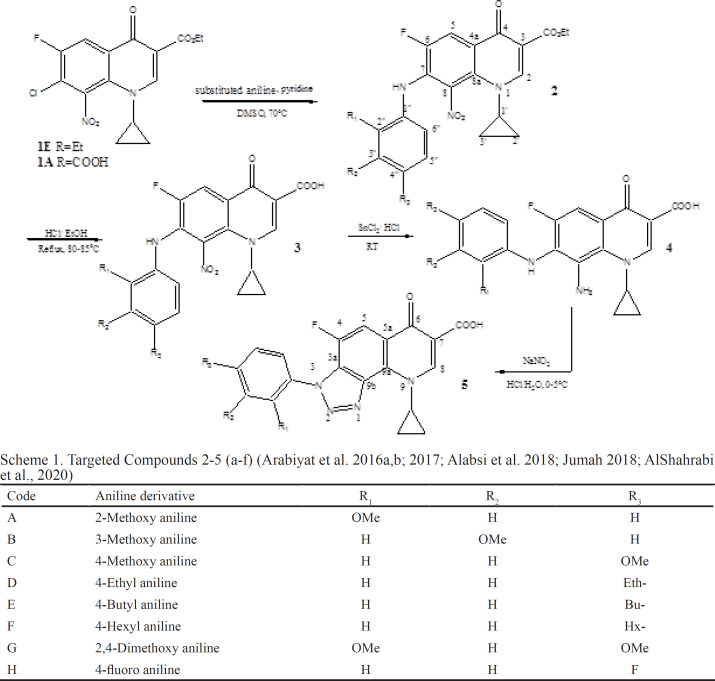

**Scheme 2:** Targeted Compounds 6a-9a (Arabiyat et al. 2016a,b; 2017;Alabsi et al. 2018; Jumah 2018; AlShahrabi et al., 2020)

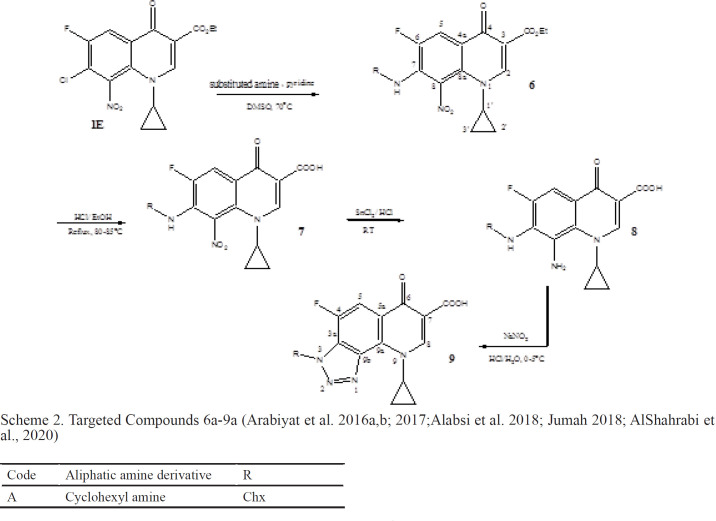

**Scheme 3:** Targeted Compounds 10-13 (a-f) (Arabiyat et al. 2016a,b; 2017;Alabsi et al. 2018; Jumah 2018; AlShahrabi et al., 2020)

Code	Aniline derivative	R_1_	R_2_	R_3_
A	2-Methoxy aniline	OMe	H	H
B	3-Methoxy aniline	H	OMe	H
C	2,4-Dimethoxy aniline	OMe	H	OMe

## Discussion

It was noticed that the reduced series with methoxy group(s) on C-7 aniline **4a**, **4b**, **12a**, **12b**, **12c** gave the best activity against K562 and PANC1. The nitro and triazolo compounds having methoxy group (**3a**, **3c**, **11a**,** 5a**, **5b**, **13b**, **13c**) have also showed strong antiprolifererative activity against same cell lines. These results indicate the need for methoxy substituents (anisidine) within the structural scaffold of these compounds. This fortifies the assumption that more H-B group does increase antiproliferative activity provided in this case from oxygen atom of the MeO group. Again, increasing lipophilicity through 4’-alkane substitution on C-7 aniline such as ethyl, butyl and hexyl in compounds **3e**, **4e**, **4d**, **4f**, **5e **have increased antiprofilerative activity significantly furnishing nano molar IC_50_ against K562, MCF7 and PANC1 cell lines. This confirms that lipophilic compounds are essential for anticancer activity mainly against these 3 cell lines. Since both N-1 alkyl groups (cyclopropyl or ethyl) have shown no significant difference in activity, this designates that lipophilicity which matters not alkane chain type on N-1. Although the nitro and reduced series showed superlative activity against A549 and PANC 1 cell lines, the triazolo activity was shifted against the two breast cell lines MCF-7 and T47D exemplified by **13c**, **5a**,** 5b**. This suggests that the extra ring imposed on triazolo derivatives has changed the mechanism of action of these FQs. It is well known that lipophilic poly cyclic system with basic amino atom(s) might work as DNA-intercalators and fit itself among the DNA grooves. The summary of SARS prediction of these FQs is proposed in Figure 2. 

*Antimicrobial activity*

This project aimed at preparing new lipophilic FQs **3-13** and investigating their antiproliferative activity against 7 cancer lines and fibroblast. The main idea is to prepare lipophilic FQs through baring N1- alkane and C-7 substituted aniline. Additional C-7-substituents (4’-anisidines and 4’alkane chains) contributed to their lipophilicity and added extra Hydrogen Bonds. 

The advantage of such modification is to allow cancer cell membrane penetration through lipophilic phospholipid bilayer which is different from active efflux pump in commercial antibacterial FQs. Contrasting to our lipophilic compounds **3-13**, most antibacterial FQ drugs in the market are hydrophilic showing superior activity against Gram-negative bacteria compared to much lower activity against Gram-positive strains. The reference ciprofloxacin showed MIC 0.0278 **µM against** Gram negative *E.coli* whereas it revealed 1.12 **µM **against Gram positive *S. aureus* -Supplementaries 4-5).

The cell wall of Gram negative bacteria has outermembrane which contains lipopolysaccharide layer. Gram positive bacteria have thick cell wall mainly composed of peptidoglycan. We do predict that due to the high lipid content in the outermembrane of Gram negative bacteria, the lipophilic FQs 3-13 (McFarland, 1907; Al-Hiari et al., 2007; Kasabri et al., 2014; Kasabri, 2017; Abdul Fattah et al., 2019; Arabiyat et al., 2020) are trapped in this membrane which reduces their activity. While in Gram positive bacteria they can pass the cell wall for an active cell penetration through the phospholipid bilayer membrane of Gram positive bacteria (Biagi et al., 1970). Since both cancer cells and Gram positive bacteria share this phospholipid bilayer mechanism, we postulate that the activity of our lipophilic FQs must be shifted to be stronger against Gram positive strain. To validate this hypothesis, we carried out antibacterial activity of our compounds against Gram positive and negative strains.

Initial high through put through our FQs 3-13 via qualitative evaluation of zone of inhibition approach has revealed mostly the superior anti-*S. aureus* *ATCC6538 *efficacy of lipophilic FQs scaffolds with matchless anti-*E. coli ATCC25922* or anti-*C. albicans* *ATCC10231 *efficacies (Supplementaries 1-3). This can be distinctive evidence with extra validation of our hypothesis in which lipophilic anticancer FQs have greater anti-Gram positive activity further revealed via minimal MICs (µM) of compounds **3-13** against *S. aureus* ATCC 6538 but not against *E. coli* *ATCC 25922* respectively vs. the reference ciprofloxacin (Supplementaries 4-5 and Figure 2). 

Supplementaries 4-5 and supplementary Figures 2-3 disclose that most FQs do have marginally appreciable antibacterial activity against Gram negative *E.coli* than against *S. aureus*. Much fewer tested compounds had any activity against E.coli with MIC values higher than their respective values against the Gram positive strain. In fact, more than 18 compounds were inactive against *E.coli*. Unlike the nitro- and triazolo- series, the reduced series exhibited the strongest antibacterial activity against both bacterial strains with exceptional activity on *S. aureus*. Remarkably Nine out of 11 reduced compounds (4 series) had MIC values <10 µM (Figure 3). Compound **4g** showed the most highly significant antibacterial activity (MIC = 0.004 µM) that is incomparably to reference against S.aureus. Similarly, compounds **4b** and **4e** revealed within nanomolar MIC with 0.024 and 0.32 µM respectively (Supplementary 4) against *S. aureus*. This obviously proposes the necessity for C-8 amino group of extra H-B contributing to distinctively enhanced anti-Gram positive activity. The results also confirm the substantial impact of 4-oxo-3-COOH ionizable acidic group as an essential side chain of the antibacterial FQ scaffold required against Gram positive strain. Conversely the **2A** and **2h** esters lacked antibacterial activity while the **1A** acid exerted incomparably appreciable activity.

*Conclusion and Future Work*

Thirty six functionalized lipophilic FQs were synthesized, characterized and tested for their anti-proliferative and antimicrobial activity. The anti-proliferative activity was tested against 7 different cancer cell lines **A549**, **A374.S2**, **MCF7**,** HELA**, **K562**, **PANC 1**, and **T47D**. Our compounds have reasonable to strong antiproliferative activity against all cell lines with superior activity against Leukaemia cell line K562. Other pronounced activity was noticeable against HELA and against PANC 1, A549, MCF7 and T47D. The antimicrobial activity was more on gram positive than on gram negative activity. The reduced series 4 with the dual action had the strongest potency among both activities. C-8 anililine lipophilic group and free acidic COOH group are assumingly associated with the functionalised FQs scaffolds with safety profiles. Future work includes screening the active compounds against other cell lines such as prostate cancer cell lines and screening the active compounds against other bacterial gram positive normal and resistant bacterial strains; Clinical testing /toxicity of active hits; Preparation of new lipophilic FQs on different position; Investigation of the action mechanism of those compound (topoisomerase II possibly) and conducting QSAR and docking study to further predict and validate new active FQs. 

## Author Contribution Statement

Authors’ contributions All authors contributed equally towards rationale conceptualisation, experimental design, data collection and analyses, manuscript write up, and proofreading 
